# Genetic diversity, population structure in a historical panel of Brazilian soybean cultivars

**DOI:** 10.1371/journal.pone.0313151

**Published:** 2025-01-30

**Authors:** Adriel Carlos da Silva, Danielle C. Gregorio da Silva, Everton Geraldo Capote Ferreira, Ricardo V. Abdelnoor, Aluízio Borém, Carlos Arrabal Arias, Marcelo F. Oliveira, Marcio Elias F. de Oliveira, Francismar Corrêa Marcelino-Guimarães

**Affiliations:** 1 Centro de Ciências Biológicas, Programa de Pós-Graduação em Genética Melhoramento, Universidade Federal de Viçosa, Viçosa, Minas Gerais, Brazil; 2 Empresa Brasileira de Pesquisa e Agropecuária-Embrapa Soja, Laboratório de Biotecnologia Vegetal e Bioinformática, Londrina, Paraná, Brazil; 3 The Sainsbury Laboratory—TSL, University of East Anglia, Norwich, United Kingdom; 4 Embrapa Recursos Genéticos e Biotecnologia, Brasília, Distrito Federal, Brazil; University of Guelph, CANADA

## Abstract

Soybean [*Glycine max* (L.) Merrill] is one of the most widely grown legumes in the world, with Brazil being its largest producer and exporter. Breeding programs in Brazil have resulted from multiple cycles of selection and recombination starting from a small number of USA cultivar ancestors in the 1950s and 1960s years. This process has led to the successful adaptation of this crop to tropical conditions, a phenomenon known as tropicalization. Many studies describe a narrow genetic background in Brazilian soybean cultivars. Various factors can affect the genetic diversity in species, especially in cultivated crops, such as the reproduction type, artificial selection, and the number and sources of variability in the breeding programs. In turns, the genetic diversity can affect the linkage disequilibrium blocks (LD) patterns and, consequently, molecular breeding strategies for selection of target loci for agronomic traits. We used high-throughput genotyping with SoySNP50K Illumina SNP markers to assess a collection of 370 Brazilian soybean accessions covering more than 60 years of soybean breeding in Brazil. Our goal was to investigate population structure and genetic diversity in the Brazilian germplasm, detect patterns of LD blocks, and identify regions presenting signals of selective swaps linked with quantitative trait loci (QTLs) of agronomic interest. Population structure analysis revealed two major groups among all genotypes, primarily differentiated by the year of release, separating old and new cultivars (before and after 2000´s years), and by growth habit (stem termination type—SST). The group I comprises about 75% of the panel and includes cultivars release before 2000`s years, including the oldest cultivars released in Brazil, most of which exhibit a determinate growth habit and maturity groups VI and VII. Group II includes only 83 materials, but shows higher levels of diversity than group I, representing most recent introductions in Brazilian germplasm. Further analysis of substructure within Group I, identified seven subgroups with no clear trend for segregation based on maturity group, STT or year of release. Instead, these subgroups were based on the contribution of key donors of disease resistance and adaptability, as soybean cultivation expanded from the South to Central region of Brazil. This finding is consistent with the history of soybean expansion in Brazil. We identified 123 genomic regions under selection among the groups of Brazilian cultivars associated with 440 quantitative trait loci (QTLs), revealing regions fixed across the breeding process associated with yield, disease resistance, water efficiency use, and others.

## Introduction

Soybean (*Glycine max* L.) is the most important crop used for animal feed and human foods because it is an excellent source of vegetable oil and protein [1]. It is a significant economic crop, accounting for more than half of global oilseed production [2]. Brazil is currently the world’s top producer, accounting for approximately 40% of global soybean production [3, 4].

Soybean was first introduced into Brazil in 1882, at a latitude of around 12° LS (state of Bahia), but it did not adapt well. New cultivars were introduced in 1891 at a latitude of around 22° 54’ LS (Campinas, SP) and in the state of Rio Grande do Sul. Soybean performed well in this southernmost state of the country due to weather conditions, primarily the photoperiod, being similar to those in the southern United States [5]. However, it was still grown on a small scale as late as 1915. As soybean became more important in Brazil, it became essential to develop cultivars adapted to its conditions. Crosses between introduced cultivars began to occur in order to get the first Brazilian varieties, yielding cultivars such as Industrial, Planalto and Santa Rosa [6, 7].

After the 1990s, with globalization and the approval of the Plant Protection Law, a new period for soybean breeding started in Brazil. This period was marked by the participation of large international corporations working to develop adapted and productive varieties, as well as the introduction of GM (genetic modified) traits. With the soybean introductions in Brazil, the average yield was 1,971.4 kg.ha^-1^ in the late 1980s and reaching to 3,275.2 kg.ha^-1^ in 2020 with modern cultivars, representing a 66% increase.

The genetic background of Brazilian soybeans background is predominantly derived from only 26 common ancestors, with just four accessions (CNS, Tokyo, Roanoke, and S-100) accounting for over half of this base [7]. The number of cultivars that make up the genetic foundation of Brazilian cultivars has remained generally constant until 2000 [8]. Although the number of common ancestors has lately increased to 60, a small group of individuals still contributes significantly to the majority of the genetic basis, categorizing the current Brazilian soybean germplasm pool as narrow [9].

Various studies on genetic diversity among Brazilian soybean germplasm have demonstrated low genetic diversity. Gwinner et al. (2017) [10] evaluated the population structure of 77 Brazilian genotypes with SSR markers, covering the years 1998–2012, and found two principal groups (K = 2), mainly segregated by early and late cultivars. Contreras-Souto et al. (2017) [11] evaluated a large set of 169 Brazilian tropical germplasm at the genomic level using BARCSoySNP6K markers, finding high genetic relatedness and a common genetic base among public and private breeding programs in Brazil. More recently, Mendonça et al. (2022) [12] compared Brazilian cultivars with those from North America and Asia using 2,175 SNPs obtained by Genotyping by Sequencing (GBS) and identified three subpopulations originating from different geographic regions. Asian genotypes were the most distinct, presenting higher genetic diversity than Brazilian genotypes. Among Brazilian samples, cultivars developed by the same company and within the same maturity group (MG) showed higher genetic similarity.

A significant genetic bottleneck during soybean domestication was also reported in numerous independent studies [10–12], demonstrating a reduction in genetic diversity and loss of valuable traits. This loss in genetic diversity is common in crops that have a small number of elite lines used for breeding future generations and that have been subjected to significant selecting pressure focused on genes influencing agronomic qualities during domestication and later episodes of selective breeding [10, 13].

Thus, assessing genetic dissimilarity between genotype pools or between individuals is a valuable tool for breeding programs. It allows for a more effective parental selection and the development of progenies with high genetic variation, potentially increasing the selection gain [14–16]. Recently next-generation sequencing methods and single nucleotide polymorphism (SNP) genotyping advancements opens a new horizon of applications in plant breeding [17–19], becoming an important tool for studying soybean diversity and evolution at genomic level. SNPs are appropriate for most high-throughput genotyping applications due to their abundance and even distribution across the genome [17]. In this study, we used high throughput genotyping with SNPs markers of BARCSoySNP50K Bead Chip Illumina [20] to assess a collection of 370 Brazilian soybean accessions spanning 64 years of intensive artificial selection on breeding programs to: (1) investigate the population structure and genetic diversity among a large set of historical Brazilian soybean cultivars, (2) detect the LD patterns, (3) identify genomic regions under selection based on population structure clusters, and (4) identify quantitative trait loci (QTLs) present in these genomic regions.

## Material and methods

### Soybean genetic data

The soybean materials used in the study were obtained from the Embrapa’s Soybean Germplasm Collection. A total of 370 Brazilians cultivars, spanning 64 years of breeding, from various maturity groups (MG), stem termination types (STT), and dates of release were employed (YEARS). DNA of the accession was extracted by DNA-easy Plant Kit (Qiagene) and submitted to analysis with the SoySNP50K iSelect Bead Chip, using the Illumina platform (Illumina Inc., San Diego, CA, USA) at Beltsville Agricultural Research Center, USDA-ARS, Beltsville, MD, USA. The hybridization data was analyzed in GenomeStudio V2011.1 software (Illumina Inc., San Diego, CA, USA). Tassel 5 [21] and vcftools [22] were used to remove SNPs showing missing data and with minor allele frequency (MAF) lower than 0.01, and heterozygosity greater than 0.10, resulting in 19,753 SNPs remaining. These SNPs were used for all analyses, except for the haplotype blocks and genome wide associations (GWAS) analysis, which utilized SNPs with MAF higher than 0.05, resulting in a total of 15,365 SNPs. The analysis were based on the soybean genome: Glyma.Wm82. a1, and only biallelic variation was used in the final panel.

### Soybean population structure and genetic diversity analysis

The Structure software v2.3.4 [23] was used to generate the population structure analysis, which included a 5,000 burn-in time and 50,000 Markov Chain Monte Carlo (MCMC) repeats for K ranging from 1 to 12. For each K, we ran 20 repetitions, and we used Structure Selector to choose the optimal delta K values based on the Evanno’s criteria. We classified the cultivars based on the findings of the Population structure study.

To access genetic diversity, we utilized the vcftools software to calculate the population fixation index coefficient (Fst) and nucleotide diversity coefficient (π). TASSEL 5 was used to calculate the linkage disequilibrium parameter (r^2^), and the LD decay graph was plotted in R software (R Development Core Team 2016), with physical distance (Mbp) vs r^2^ using nonlinear regression as reported by [24]. TASSEL 5 was also used for principal component analysis (PCA) and to create a dendrogram utilizing the UPGMA (unweighted pair group method with arithmetic mean) algorithm.

The distribution of genetic diversity within and between populations was obtained through the Molecular Variance Analysis (AMOVA), performed according to [25], with the packages poppr and ade4, aided by the R package vcfR. The R package poppr and STAMPP were used to calculate Nei genetic diversity [26] between the groups and between individuals. The total percentage of heterozygosity (%Het) and the average MAF were determined with Tassel 5.

### Haplotype blocks analysis

LD blocks were determined using Plink 1.9 [27]. Plink 1.9 identified haplotype blocks by estimating D’ for all pairwise SNPs combinations within 1 Mb windows and calculating LD (r^2^) across SNPs to identify haplotype blocks, following the block concept interpretation presented by [27]. Pairs of SNPs were in a haplotype block if the one-tailed upper 95% confidence limit on D’ was higher than 0.98 and the lower limit was greater than 0.7 [28].

### Identification of selection signatures in the soybean Brazilian panel

The Fst-outlier technique implemented in the BayeScan program [29] was used to detect selection signatures between the two main groups of accessions revealed by population structure analysis. This statistical tool identifies loci under selection by comparing their Fst coefficients to expected values. Significant genetic differences (high Fst) are expected between populations when genes are subject to local directional selection, and similar allele frequencies (low Fst) when genes are under balancing or purifying selection across populations. Fst coefficients were partitioned using logistic regression in BayeScan, allowing access to a locus-specific component (α) shared by all populations. When α is significantly different from zero, it indicates selection; when it is greater than zero, it implies directional selection; and when it is below zero, it indicates balancing or purifying selection.

BayeScan analyses consisted of 20 pilot runs of 50,000 iterations each, followed by 100,000 iterations on a 5,000-sample size with a thinning interval of 10. Prior odds were set to 10. Outlier loci were detected using BayeScan [29] by selecting SNPs with posterior odds thresholds equal to or higher than 1 (PO ≥ 1), leading to a false discovery rate of no more than 5% (FDR ≤ 0.05).

### Identification of quantitative trait loci associated with genomic regions under selection

We used Soybase QTL data to find QTLs associated with genomic regions under selection. All SNPs as under selection by outlier method in BayeScan were examined. A SNP was considered linked to a QTL’s if it was inside the identified haplotype blocks. The SNPs under selection and associated QTLs distribution on haplotypes blocks in the soybean chromosomes were presented in the Circus Plot [30].

## Results

### Soybean panel characterization

**Fig 1:**
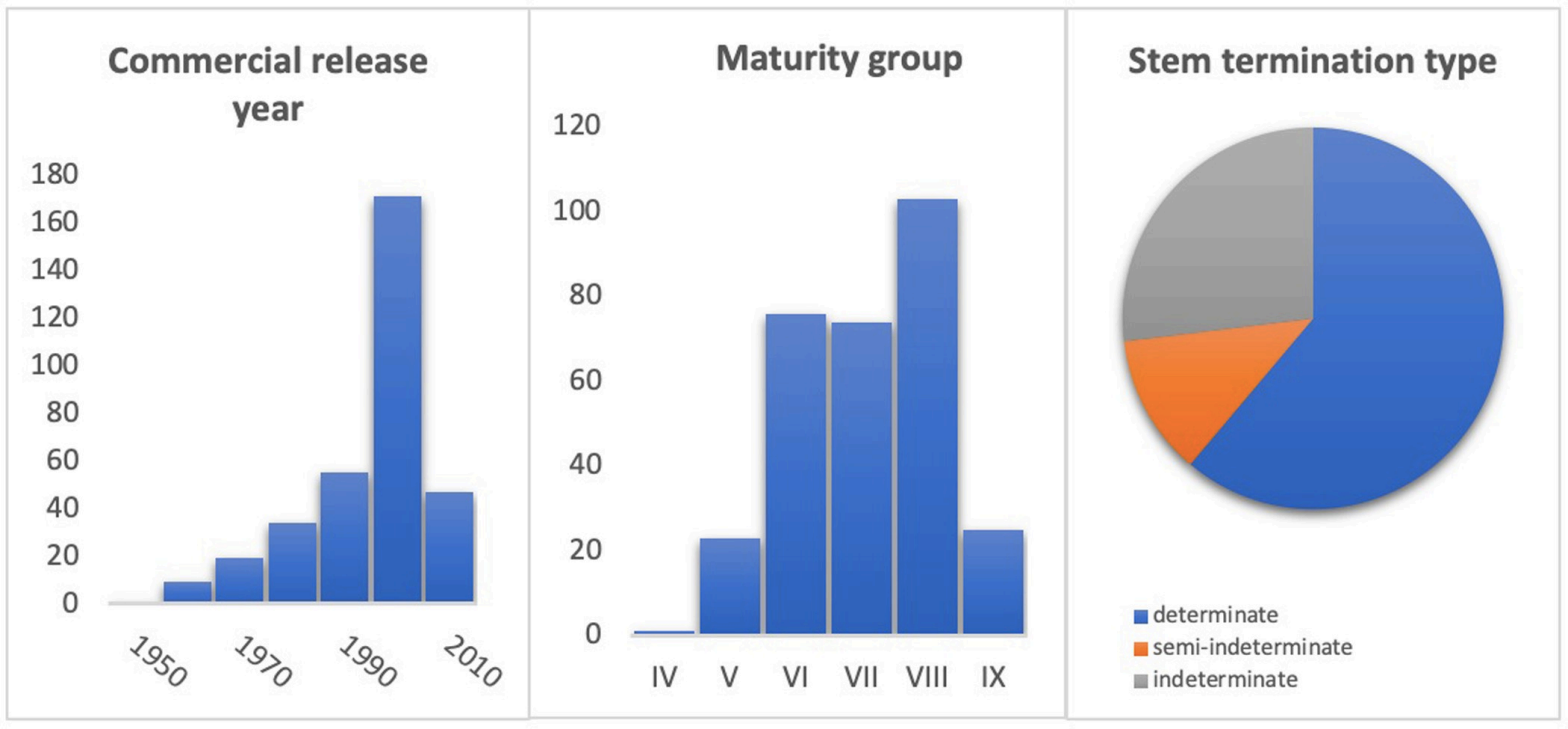
Characterization of soybeans cultivars in the panel based on (a) commercial release year by decades (336 samples); (b) maturity group (302 samples); (c) stem termination type (260 samples).

The soybean panel used in this study consists of 370 cultivars from many companies, primarily from EMBRAPA (Empresa Brasileira de Pesquisa Agropecuária), covering release years from 1952 to 2016 (S1 Table). These accessions include both Brazilian soybean cultivars available on the market and introduced cultivars that have served as important ancestors for Brazilian soybean lines. Three Japanese cultivars were also included in the set as important donors of resistance genes to soybean rust in Brazilian germplasm recently. The distribution of the cultivars based on the year of release shows that approximately one-third of the panel (118 cultivars) was released before 2000, mostly of them concentrated in the first decade of 2000´s years (171), while a set of 47 cultivars released after 2010 (Fig 1A). Because these lines were designed for various Brazilian regions, they have a wide range of MG, ranging from MG IV to IX. (Fig 1B). Regarding STT, the panel comprises 61.2% as determinate, 11.9% semi-indeterminate and 26.9% indeterminate (Fig 1C).

### SNP distribution in Brazilian germplasm

Of the 51,955 SNPs in the SoySNP50K dataset, 19,576 SNPs remained after filtering by MAF >1%, heterozygous count <10% and no missing data. This includes 16,688 SNPs (85%) in the euchromatic and 2,888 (15%) in the heterochromatic regions (third track of Fig 6), distributed in 1,753 LD blocks (Table 1). These high-quality markers were used in the subsequent analyses. The 19,576 SNPs presented an average of 979 SNP per chromosome, ranging from 616 on chromosome 20 to 1,575 on chromosome 18. After the filtering, the average MAF value was 0.20, with a median of 0.168 and a standard deviation of 0.154.

**Table 1:** SNPs marker distribution and linkage disequilibrium blocks in Brazilian soybean cultivars over 20 chromosomes (Chr).

Chr	Length Chr (Kb)^a^	Blocks in Disequilibrium^b^	Blocks’ Length (Kb)^b^	Number of SNPs	Average marker density SNP/kb	Number of SNPs in LD Blocks	Average Density SNPs/Block
1	56832	91	23640	993	57.232	834	9.2
2	48578	104	18499	1088	44.648	894	8.6
3	45780	80	15804	797	57.440	623	7.8
4	52389	76	24137	907	57.761	728	9.6
5	42234	70	11538	935	45.171	761	10.9
6	51416	94	19555	1052	48.875	786	8.4
7	44631	77	19845	953	46.832	751	9.8
8	47838	97	22970	1125	42.523	947	9.8
9	50190	86	23830	1051	47.754	872	10.1
10	51567	74	17800	915	56.357	734	9.9
11	34767	78	19252	948	36.674	829	10.6
12	40091	63	13077	761	52.682	635	10.1
13	45874	133	18851	1222	37.540	1066	8.0
14	49042	88	22413	779	62.955	636	7.2
15	51756	107	30228	1176	44.010	1040	9.7
16	37887	96	13455	968	39.139	715	7.4
17	41641	76	19760	929	44.824	723	9.5
18	58019	125	38485	1575	36.837	1353	10.8
19	50747	69	20368	786	64.564	696	10.1
20	47904	69	12482	616	77.767	488	7.1
**Total**	**949,183**	**1,753**	**405,990**	**19,576**	**50,079** ^*^	**16,111**	**9.2** ^*^

^a^ Source Soybase (www.soybase.org); ^b^ haplotype block analysis based on 15,365 markers (MAF 5%); * Average.

As expected, most of the SNPs were homozygous. The percentage of heterozygosity ranged from 0.03% in the BRS 246 RR to 52% in the variety TK5, with a mean of 0.52% among the 370 cultivars. Approximately 99.4% of the varieties (368) had fewer than 10% of heterozygosity. The only two varieties which had more than 10% of heterozygosity were TK-5 and GB 881 RR (10.01%).

### Soybean population structure and genetic diversity

**Fig 2:**
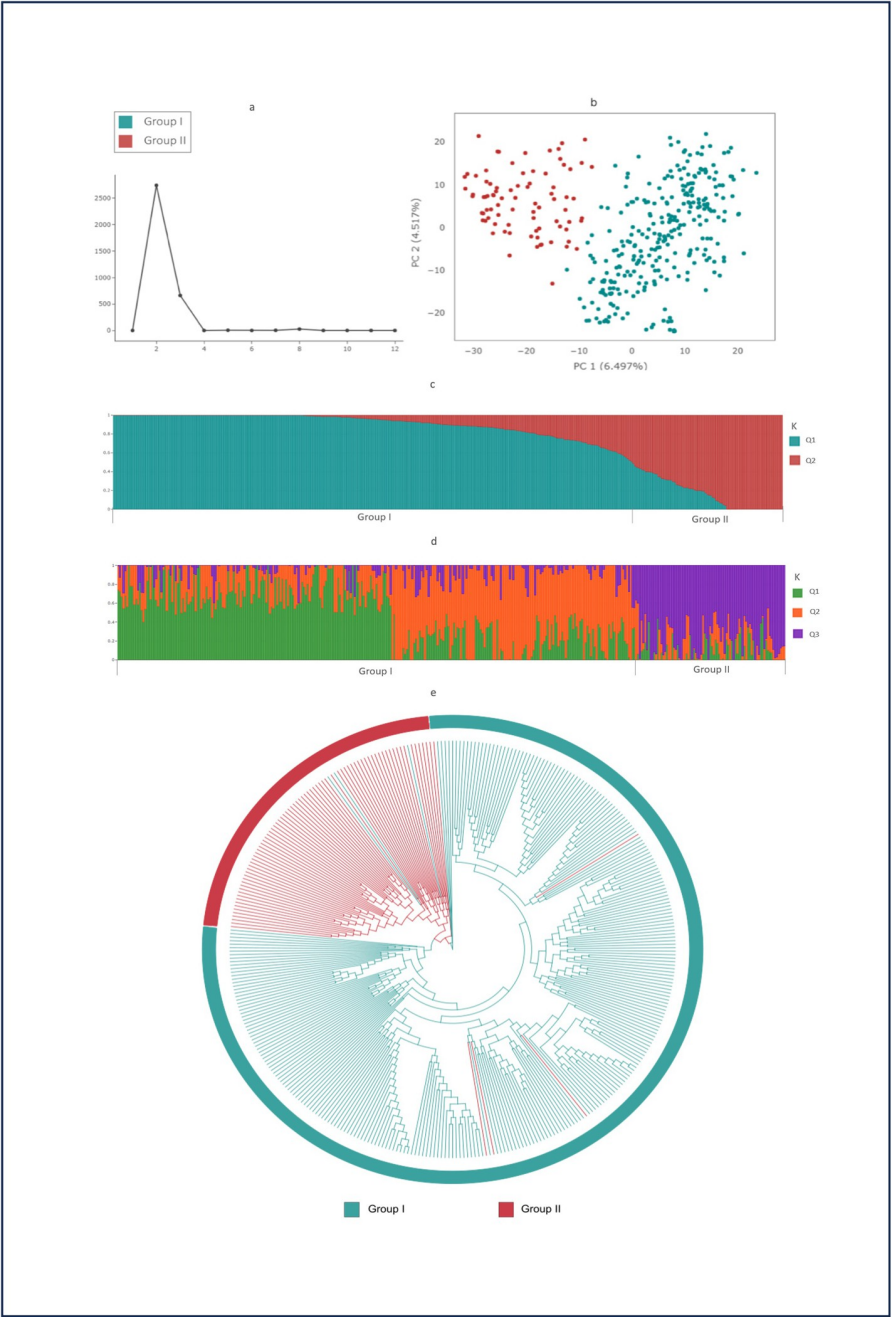
Population structure and phylogenetic tree of a set of 370 Brazilian soybean cultivars evaluated with 19,576 SNPs; (a) Delta K-values for k = 1–12; (b) Principal component analysis (PCA) plot; (c) Bar plot of individual cultivars considering K = 2. Group I (blue) with 287 cultivars and Group II (red) with 83 cultivars; The colors represent the grouping as determined by population structure analysis; (d) Bar plot of individual cultivars considering K = 3. Each individual is represented by a single vertical line with lengths proportional to each of the inferred clusters; (e) UPGMA Phylogenetic tree inferred from whole-genome SNPs of all accessions. The colors represent the grouping as determined by population structure analysis K = 2.

Different complementary approaches, such as STRUCTURE, UPGMA clustering, and principal component analysis (PCA), were used to describe the population structure of the soybean panel. According to the Evanno criteria (Fig 2A), the optimal number of groups (K) was determined to be 2, with the highest ΔK value of 2,745.55. However, a three-group (K = 3) structure also had a high ΔK value of 660.65. Thus, K = 2 was considered the best value to explain the population structure of the panel. Each subpopulation consisted of individuals with varying genetic backgrounds (Fig 2C). Group I included 287 genotypes, while Group II comprised 83 genotypes. Each subpopulation (K = 2) encompassed admixed cultivars from various Brazilian soybean genetic breeding programs and maturity groups.

Principal component analysis was also used to assess the population structure (Fig 2B). The first two major components explained 6.5 and 4.52% of the total variation, respectively. The genotype distribution suggested the establishment of two clusters, as proposed by the population structure analysis.

The phylogenetic tree revealed a similar clustering pattern to that observed in the PCA and population structure analyses, identifying two main branches (Fig 2E). One branch contained 81 cultivars, while the other branch included 284 cultivars (S1 Table). The clustering patterns from the population structure and phylogenetic tree analyses provide insights into the development and introduction of current elite soybean cultivars. Additionally, some cultivars were found to have mixed ancestry, indicating that these lineages may have undergone introgression or gene flow during breeding.

The average Fst value between the two populations was 0.1074. The different subgroups exhibited variations in nucleotide diversity (π) and population structure. The average π among genotypes in Group I was slightly lower than in Group II, with values of 0.261 and 0.275, respectively. The average Nei genetic diversity was 0.3015 for Group I and 0.3226 for Group II, resulting in a Nei genetic diversity difference of 0.0570 between the two groups (Table 2).

**Table 2:** Intrapopulation genetic diversity in groups I and II of Brazilian cultivars.

Populations	N	%Het	Nei	π
Group I	287	0.00499	0.3015233	0.261
Group II	83	0.006	0.3225912	0.275
	370			

**N**. is the number of individuals; **%Het**. is the percentage of heterozygosity; **Nei.** Is the average Nei genetic diversity; **π**. is the nucleotide diversity coefficient.

AMOVA revealed that only 12.84% of the molecular variance was due to the genetic distance between the two clusters, while 87.16% of the variation was among individuals within populations (S2 Table).

**Fig 3:**
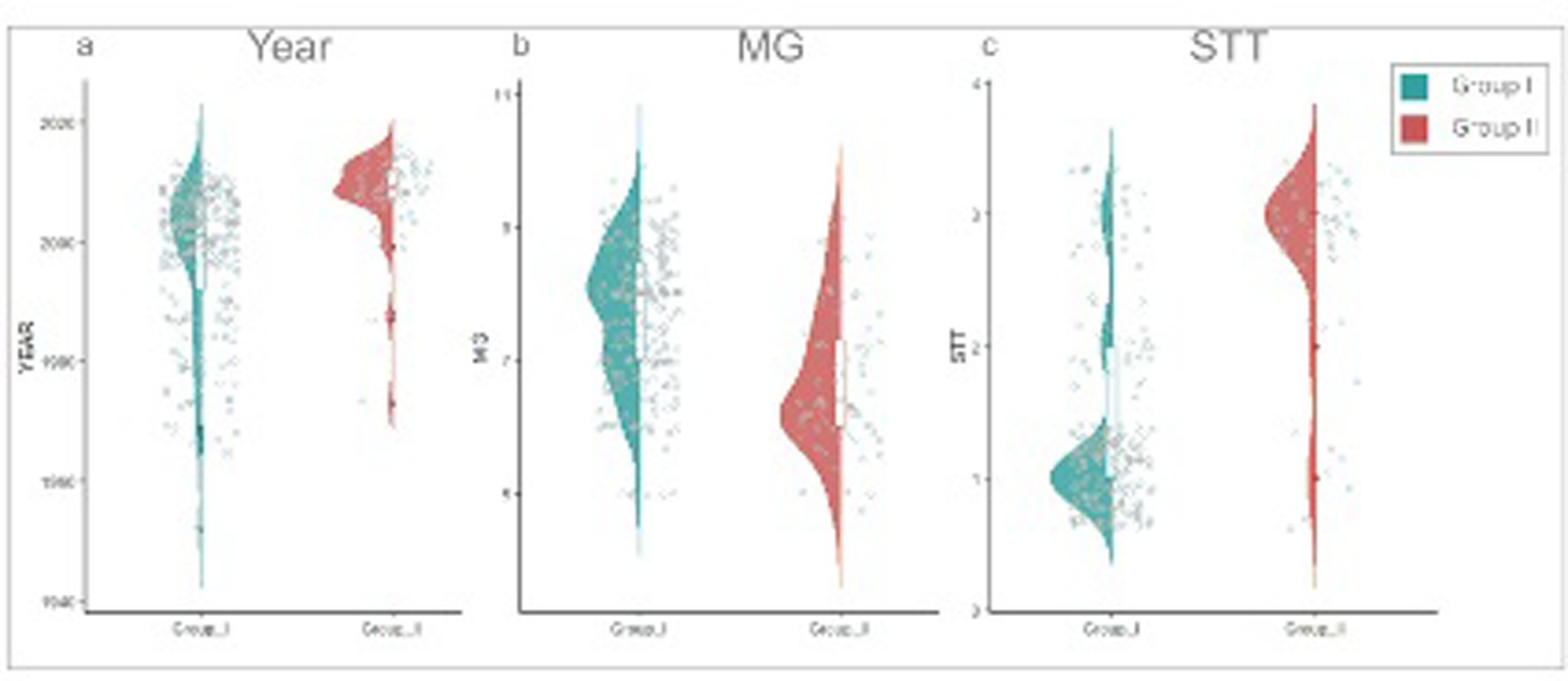
Distribution of the Brazilian soybean cultivars in Groups I and II considering (a) Year of release (336 accessions); (b) Maturity group (302 accessions) and (c) Stem termination type (260 accessions).

For K = 2, we identified three distinct grouping patterns that may have influenced the grouping trend. First, cultivars primarily belonging to the same stem termination type were clustered together in group II, with determinate cultivars showing a higher degree of genetic similarity among themselves. Second, materials developed primarily after 2000 were also placed in Group II. Third, the maturity group (MG) was slightly higher in Group I compared to Group II. When the two groups were compared by MG (N = 302), release YEAR (N = 336), and SST (N = 260), the correlations were -0.391, 0.383, and 0.614, respectively. The averages values for groups I and II were VII and VI for MG, 1997 and 2008 for year, and 1.4 and 2.7 for STT (Fig 3).

In Group I, we highlighted some of the oldest materials developed in Brazil during the 1950s and 1960s, such as the cultivars Pelicano (1951), Pioneira (1960), Santa Rosa (1965), and IAC2, 3, 4, and 5 (1966), Industrial (1967) and Vicoja (1969). This group also includes some North America cultivars adapted from the southern United States that were introduced in Brazil, such as CNS, Hardee, Bragg, and Davis. Additionally, the first cultivars developed by Embrapa and IAC (Instituto Agronômico de Campinas) are also in Group I, including BR1 (1976), BR4 (1979), IAC2 (1967), and IAC4 (1975).

In Group II, we observed that the majority of the cultivars were released after 2000’s years (77 out of 83), such BRS284, BRSGO-8360, BRS1003-IPRO, BRS-7380-RR released by Embrapa, as well as materials from other companies, such as Brasmax (BMX-Apolo, Potencia, Desafio and V-Max-RR), TMG–Tropical Melhoramento Genético (TMG 108, 1180, 1188), and Coodetec (CD 216, 225RR, 240RR), among others. These materials were highly adopted cultivars by farmers presenting high yield adaptability, and a tendency for a short cycle.

### Group I sub-structure and genetic diversity

**Fig 4:**
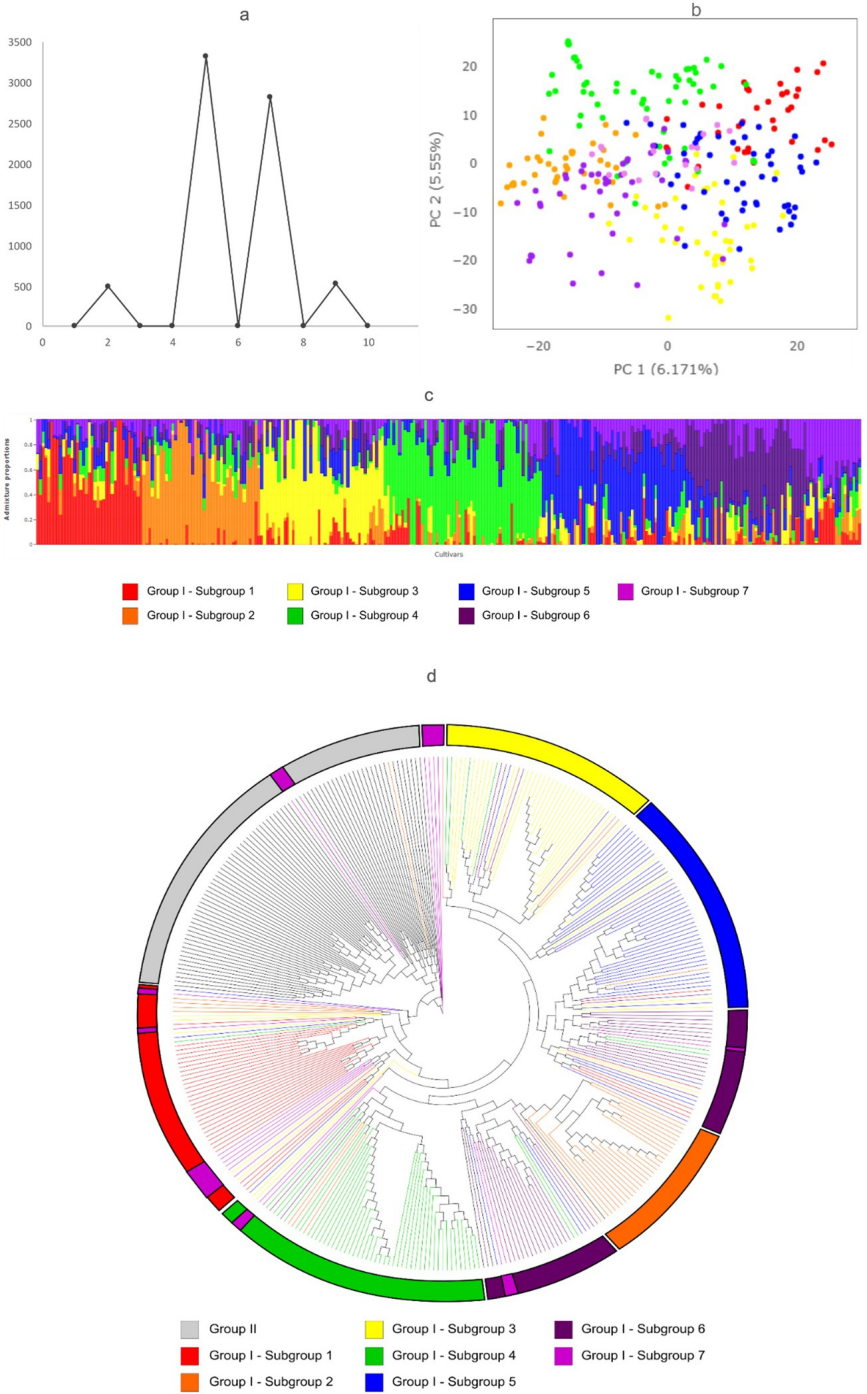
Population structure of the Group I, composed of 287 Brazilian soybean cultivars evaluated with 19,576 SNPs; (a) Delta K-values for k = 1–12; (b) Principal component analysis (PCA) plot; (c) Bar plot of individual cultivars considering K = 7. The colors represent the grouping as determined by population structure analysis; Each individual is represented by a single vertical line with lengths proportional to each of the inferred clusters; **(d)** UPGMA Phylogenetic tree inferred from whole-genome SNPs of all accessions. The colors represent the grouping as determined by population structure analysis K = 7 with 287 accessions of Group I.

To scrutinize the panel for additional sub-structure variations within Group I (287 accessions), we repeated the populational structure analysis for this group. According to the Evanno criteria (Fig 4A), the structure findings based on seven groups (K = 7) had a high ΔK value (2,825.01), while the upper-most level of the structure was in five groups (K = 5; ΔK = 3,322.77). Thus, K = 7 was identified as the optimal K-value to explain the population structure of Group I Each subpopulation held individuals with varying genetic backgrounds (Fig 4C). Principal component analysis was also conducted to assess the population structure (Fig 4B). The first two major components explained 6.17 and 5.55% of the total variation, respectively.

The phylogenetic tree exhibited a clustering pattern similar to that observed in the PCA and population structure analyses for the seven subgroups (Fig 4D). The subgroups located at the extremities of the PCA plot showed more defined structures in the phylogenetic tree. Subgroups 1, 2, 3, 4, and 5 are grouped into distinct branches of the tree, though some genotypes from these subgroups are dispersed across the tree. Subgroup 6 is positioned in two different areas of the tree, with subgroup 2 located between them. Subgroup 7 forms a small branch of its own, but most of its genotypes are dispersed among the branches belonging to subgroups 1, 4, 6, and those of Group II. The subgroups exhibited variations in genetic diversity. According to Nei’s genetic diversity and Fst values between the subgroups (Table 3), subgroups 5 and 7 have the highest average Nei’s genetic distances to other subgroups (0.035 and 0.036, respectively), while subgroup 4 has the lowest average Nei’s genetic distance to other subgroups (0.019). The smallest Nei’s genetic distance is between subgroups 5 and 6 (0.008), while the largest is between subgroups 1 and 7 (0.056). Regarding Fst values, the highest average Fst value is observed for subgroup 5 (0.091), whereas the lowest average Fst value is for subgroup 4 (0.033). The smallest Fst value is between subgroups 5 and 7 (0.002), and the largest is between subgroups 1 and 5 (0.128).

**Table 3:** Matrix of Nei’s Diversity (Upper triangular) and FST values (Lower triangular) between subgroups of group I.

	Subgroup 1	Subgroup 2	Subgroup 3	Subgroup 4	Subgroup 5	Subgroup 6	Subgroup 7
Subgroup 1	-	0.019	0.032	0.028	0.053	0.046	0.056
Subgroup 2	0.034	-	0.017	0.018	0.043	0.037	0.043
Subgroup 3	0.070	0.027	-	0.016	0.040	0.038	0.042
Subgroup 4	0.059	0.029	0.025	-	0.018	0.015	0.019
Subgroup 5	0.128	0.099	0.092	0.034	-	0.008	0.012
Subgroup 6	0.107	0.081	0.084	0.022	0.004	-	0.014
Subgroup 7	0.119	0.082	0.079	0.019	0.002	0.003	-

AMOVA for the seven subgroups of group I revealed that 5.68% of the molecular variance was due to the genetic distance between the clusters, while 94.32% of the variation was among individuals within populations (S2 Table).

For K = 2K (Groups I and II), when examining the K = 7K sub-structure groups, we did not observe a clear trend in grouping. The seven groups were compared by maturity group (N = 234), release year (N = 269), and stem termination type (N = 204), with correlations of -0.266, -0.010, and 0.176, respectively.

Our collection spans MG IV to IX and exhibits an admixture population structure among the K = 7 subpopulations. Although the correlation of maturity group was the highest (r = -0.266) among the characteristics studied, we could not observe a clear segregation based on MG (Table 4). Among the seven subpopulations, none comprises individuals exclusively from a single MG, release year or decade, or company.

**Table 4:** Distribution of cultivars in each subgroup based on population structure of group I and maturity groups of Brazilian soybean cultivars.

MG	Clusters of Group I (K = 7)						
	1	2	3	4	5	6	7
V	0	1	1	0	0	7	0
VI	4	2	9	7	17	2	6
VII	4	5	17	13	7	7	7
VIII	16	25	7	22	11	12	2
IX	8	1	1	4	6	3	0

### Linkage disequilibrium decay and haplotypes blocks

**Fig 5:**
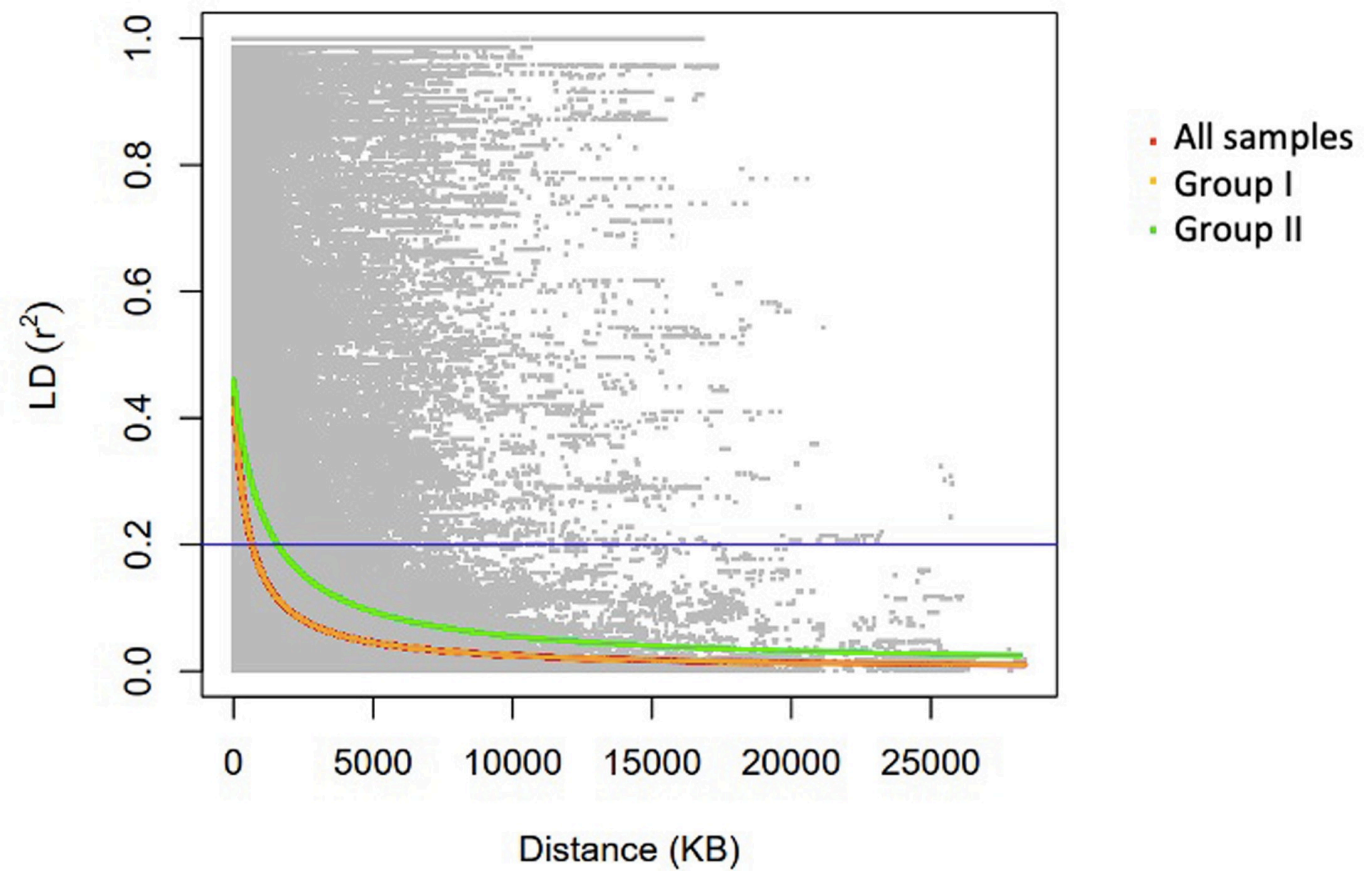
Linkage disequilibrium decay in all samples (red line), group I (yellow line) and group II (green line) 1,206.19 kb.

For all samples, linkage disequilibrium (LD; indicated by r^2^) declined to half of its highest value at 528.17 kb (Fig 5), with differences between populations. The LD declined at 517.69 kb in group I and 1206.19 kb in group II.

**Fig 6:**
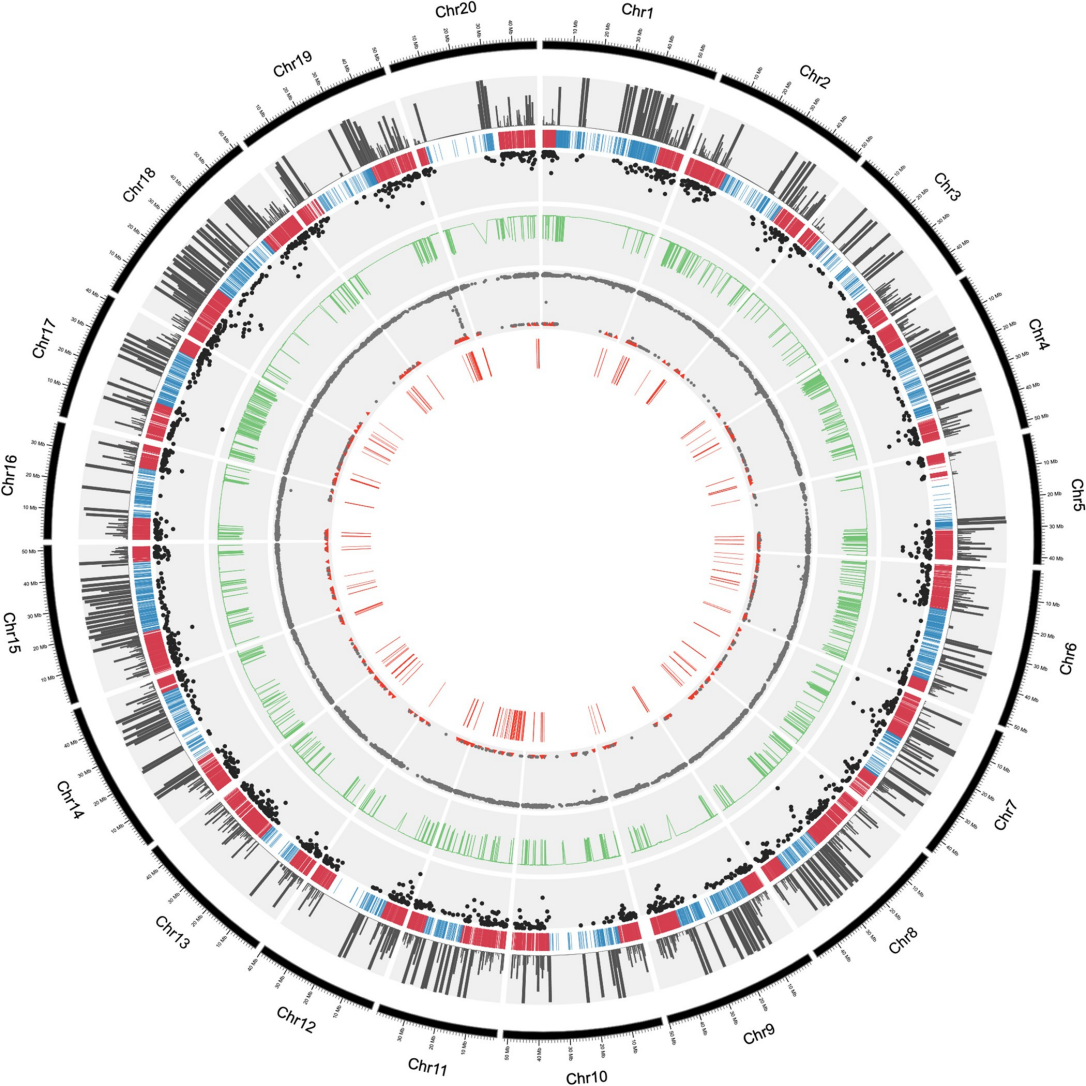
Circus plot representing the haplotype blocks distribution on soybean chromosome regions, SNP number per block, FST values, SNPs under selection and associated QTLs. Considering the concentric circles from the outside inward, the outermost (and first) black circle indicates the numbered chromosomes. The second track is a histogram of the physical position of the 1753 haplotype blocks and its sizes in kb. The third track is the physical position of the 19,576 SNPs and the chromosomes regions of Euchromatin (red) and heterochromatin (blue). The fourth track shows the number of SNPs present in each of the haplotype blocks. The fifth track shows de FST. The sixth track shows de posterior odds (PO) of the SNPS and the 547 SNPs under selection between the group I and group II genotypes (red triangle). The seventh and the inner most track shows the distribution over the chromosomes of the 440 QTLs associated to SNPs under selection.

The Haplotype block analysis, utilizing 15,365, filtered SNPs from SoySNP50K with 370 Brazilian soybean cultivars identified 1,753 LD blocks (Table 1). The blocks ranged in size from approximately 0.21 to 1 Mb, with blocks in euchromatic regions generally being smaller (see second and third tracks of Fig 6). The number of SNPs per linkage disequilibrium block ranged from 2 to 80, with an average of 7.71 SNPs per block. Approximately 80% of the LD blocks contained 10 or fewer markers (see fourth track of Fig 6). The proportion of blocks with lengths greater than 500 kb was relatively low (16.3%). The average block length was 231.6 kb. The total length of all LD blocks combined was 405,990.22 kb, representing 37% of the soybean genome’s total length of 1.1 Gb.

### Identification of signatures of selection in Brazilian cultivars

BayeScan was used to identify selection signatures across Brazilian genotypes from two separate populations (Group I and Group II), as previously determined in the structural analysis (k = 2). We analyzed 19,576 SNPs across 370 accessions, with 287 were Group I cultivars and 83 were Group II. Using the outlier approach, we found 854 with posterior odds equal to one (PO = 1) among 1,227 SNP outliers [28]. Of these 854 SNPs, 547 were distributed across 293 blocks on all 20 chromosomes (S3 Table).

We examined the genomic regions of each SNP under selection within the haplotype blocks and identified 440 quantitative trait loci (QTLs) in 123 blocks linked to 229 SNPs under selection between contrasting Brazilian cultivars in Group I and Group II. These genotypes may reflect the adaptation of the germplasm to tropical conditions through artificial. The distribution of SNPs under selection and QTLs across soybean chromosomes can be visualized in the sixth and seventh tracks of Fig 6 and S3 and S4 Tables. We identified QTLs related to four main categories: Disease Resistance, Nutrient Content and Utilization, Plant Growth and Development, and Seed Characteristics (Table 5). In the category Disease Resistance, we identified 53 QTLs distributed in 14 LD blocks, associated with resistance to fungi and oomycetes (Diaporthe stem canker, Phytophthora, Sclerotinia, and Fusarium), nematodes (soybean cyst nematodes) and viruses (soybean mosaic virus, tobacco ringspot virus and yellow mosaic virus). These pathogens are present in Brazilian conditions, affecting soybean plants survival and being targets of artificial selection during adaptation to tropical environments. In nutrient content and utilization, we identified 85 QTLs related to Nutrient Use Efficiency (e.g. B, P, K, Zn) among others, chlorosis associated with iron deficiency, and ureide content, distributed across 32 LD blocks. These QTLs may reflect the adaptation of the culture to Brazilian soils. In Plant Development and Growth category, we found 167 QTLs distributed in 58 LD blocks, highlighting QTLs related with yield (e.g., node number, pod number, seed yield and total seed number), cycle (e.g., first flower, reproductive period, R8 full maturity), plant stem termination type, and tolerance to lodging. Finally, we found 106 QTLs related to Seed Characters, distributed in 56 LD blocks. These include QTLs associated with oil quality and composition (e.g., seed oil, linoleic acid, palmitic acid, oleic acid), amino acid composition, coat color, isoflavones, and protein content.

**Table 5:** QTL categories which SNPs under selection were associated across the 20 soybean chromosomes discovered by comparing Brazilian soybean cultivars from groups I and II.

QTL Categories	QTL	Chromossome	Linked Block	# associated SNP`s
**Disease Resistance**	Diaporthe stem canker	14	1137	4
	Mexican bean beetle	11	889	1
	Phytophotora	17, 10	1465, 820	13
	Sclerotinia	11, 5, 13, 14	889, 386, 1052, 1123, 1191	13
	Soybean cyst nematode (SCN)	8, 11, 13, 16, 17, 18	1494	16
	Soybean Sudden Death (SSD)	7, 8	574	13
	Soybean mosaic virus (SMV)	12, 14	990	7
	Tobacco ringspot virus	2	157	7
	Yellow Mosaic Virus	17	1465	6
**Nutrient Content and Utilization**	Iron deficiency chlorosis	13, 18, 19	1084. 1658, 1538	5
	Leaf carotenoid content	4	322	1
	P use efficiency	1, 14, 18	59, 1196, 1568	5
	Shoot nutrients B, Ca, Cu, Fe, K, Mg, P, S, Zn	2, 4, 5, 6, 8, 9, 11, 14, 15, 18, 19, 20	186, 300, 328, 330, 328, 406, 489, 603,648, 889, 890, 741, 760, 769, 1167, 1491, 1594, 1435,1647	42
	Ureide content	2, 4, 10, 11, 13, 15, 18, 20	156, 186, 299, 820, 889, 1034, 1258, 1584, 1695	18
**Seed quality and composition**	Seed amino acid composition (Arg, Asp, Glu, His, Ile, Leu, Lys, Phe, Ser, Thr, Trp Val)	1, 6, 7, 13, 14, 15, 17, 20	75, 426, 471, 607, 703, 889, 1097, 1167, 1239, 1434, 1435, 1744	25
	Seed alpha-linolenic acid	14	1195	1
	Seed coat color	8, 15	607, 1239	3
	Seed isoflavone	1, 2, 4, 6, 15, 18	59, 147, 166, 287, 300, 474, 1298, 1584	9
	Seed protein	5, 6, 14, 11, 15,	378, 472, 889, 1124, 1239, 1262	15
	Seed thickness	2	187	1
	Seed weight	2, 3, 4, 10, 11, 12, 13, 17, 19	138, 243, 817, 889, 947, 1097, 1434, 1656, 1663, 1667	30
	Seed linoleic	13, 17, 19	1084, 1661, 1434	1
	Seed oil	5, 8, 11, 14, 17, 18	378, 597, 889, 1190, 1195, 1434, 1510, 1569	14
	Seed oleic	4, 13, 17, 19, 20	305, 1084, 1434, 1445, 1661, 1695	13
	Seed palmitic	6, 8, 14, 15, 20	433, 607,1196, 1271, 1661,1695	19
**Plant Growth and Development**	Canopy cover	1, 5	75, 406	4
	First flower	2. 3, 5, 6, 11, 12, 13, 14, 15, 16, 19, 20	138 147, 243, 354, 472, 881, 889, 890, 900, 947, 960, 1034, 1001, 1190, 1287, 1343, 1630, 1663, 1741, 1749	38
	Interbranch length	19	1667	8
	Leaflet area	19	1667	8
	Leaflet chlorophyll	13, 18	1101, 1084, 1513, 1578	4
	Leaflet width	19	1667	8
	Lodging	19	1667	8
	Node number	11, 19	889, 1664, 1667	11
	Plant height	11, 12, 13, 16, 19	889, 928, 960, 1121, 1343, 1665, 1372, 1663, 1665, 1664, 1667	25
	Pod number	6, 11, 18, 17, 19	472, 889, 1445, 1664, 1667	19
	R8 full maturity	2, 3, 6, 10, 11, 13, 16, 19, 20	138, 243, 472, 837, 889, 1084, 1330, 1667, 1749,	23
	Reproductive period	2, 4, 8, 10, 11, 19, 20	138, 193, 317, 601, 823, 889, 1667, 1749	18
	Seed set	2, 5, 9, 11, 12, 19	187, 393, 702, 881, 889, 947, 1664, 1667	26
	Seed yield	3,11, 12	243, 889, 947	7
	Stem termination type	19	1664, 1667	2, 8
	Total seed number	19	1667	8

## Discussion

### Brazilian modern cultivars present a higher diversity within a historical panel

In this study, a large set of 370 Brazilian cultivars, covering around 60´s years of genetic breeding, was analyzed at the genomic level. Consistent with other studies using various throughput technologies and datasets, we also observed the low genetic diversity in soybean Brazilian germplasm. We found that the cultivars clustered into only two main genetic groupings (K = 2) based on population structure analysis using three complementary approaches: STRUCTURE, UPGMA clustering tree, and principal component analysis (PCA) methods, rather than three groups (Fig 2A). When comparing K = 2 and K = 3, it was evident that the third group in K = 3 was essentially the same as the second group in K = 2, indicating that the first group in K = 2 was further subdivided into two parts when using K = 3. In the UPGMA phylogenetic tree, 78 of the 83 group II cultivars were clustered together in a small branch, while 280 of the 287 group I cultivars were clustered in a larger branch (Fig 2E). The same two groups were also observed in the principal component analysis, which aligned with the population structure analysis (Fig 2B). The formation of only two groups suggests low genetic diversity among the assessed soybean genotypes.

Previous soybean population structure studies with different germplasm sources (Brazilian, European, Korean, Chinese and Indian soybean, lines adapted to sub-saharan Africa, and the USDA Soybean Germplasm Collection) utilizing SSR and SNP markers identified between two to nine genetic grouping [14, 31–42]. However, in studies focusing on Brazilian cultivars, reports identified 9 [11], 3 [12, 41], and 2 [10, 31] major groups. Prioili et al. (2013) [31] also found two groups (K = 2) defining the genetic structure of a core set of Brazilian soybean cultivars, composed of 435 samples analyzed with 27 SSR markers. AMOVA analysis among the clusters revealed that 93.27% of the variation occurred within the clusters, while only 6.73% was attributed to variation among clusters. Recently, Mendonça et al, (2022) [12] observed low genetic diversity in Brazilian germplasm when compared with North America and Asian germplasms. Asian genotypes were the most distinct group compared to the rest of the panel, presenting higher genetic diversity than the Brazilian genotypes.

Similarly, studies on wild soybean also found two groups (K = 2). A broader study covering a total of 604 wild soybean accessions from 43 locations sampled across its range in China and Japan, evaluated using 20 nuclear (nSSRs) and microsatellite markers, revealed that 6.0% of genetic variation was due to the genetic distance between the two clusters, 46.7% among populations within clusters, and 47.3% between individuals within populations. These findings demonstrated that modern breeding limits gene flow, resulting in substantial variation within groups rather than between inferred groups [43, 44].

In the current study, STT and year of release were the key variables of population structure within the soybean population. Determined type, characterized by terminal and axillary racemose inflorescences with vegetative growth ceasing after flowering, belongs to the maturity group V to X, and typically grown in the Southern of the US and in Brazil. Indeterminate types, with only axillary inflorescences and classified as maturity groups 00 to IV, are primarily grown in the northern US and southernmost states of Brazil, or in central Brazil where early maturity is desired [5]. The indeterminate type was initially used in low latitudes in South America due to its similarity to varieties in the northern US, and was commonly used in Argentina before becoming more prevalent in Brazil.

Year of release and STT may be associated, since mostly of the indeterminate cultivars in this study were released after 2000 (Fig 3). The significant introduction of indeterminate materials has begun in 2000/2001, when illegal Roundup Ready (RR) soybean germplasm was smuggled into Brazil from Argentina, where it was commercially released while still illegal in Brazil [1]. Notably, Argentine company GDM (Grupo Don Mario) entered the Brazilian market in 2000, offering new germplasm. Almost all GDM cultivars were assigned to group II (S1 Table). Although maturity group did not appear to be a key factor driving the grouping pattern, there was a slight difference in the average maturity group, with group I having a higher average (VII) and group II having a slightly lower average (VI). Previous studies have highlighted the impact of maturity group on population structure [10–12, 32, 41, 42, 45].

The average Fst value (0.1074) between Groups I and II was slightly lower compared to comparisons involving more diverse sets of cultivars, such as Brazilian versus Asian cultivars, but higher than the Fst observed among Brazilian cultivars from different companies [12]. For example, [46] found an Fst value of 0.2317 when comparing wild genotypes to landraces, and 0.1060 when comparing landraces to North American cultivars. [41] observed high Fst values associated with high nucleotide diversity (π) in older (pre-1980) Brazilian cultivars compared to more recent (post-2000) cultivars, confirming differences between the two groups and suggesting the presence of sub-regions under positive selection in Brazilian soybean cultivars. The Fst values found in this study indicate a high level of genetic similarity among the 370 tested varieties, likely due to artificial selection during breeding.

Although Group II contains less than one-third of the genotypes of Group I, it exhibited higher genetic diversity according to Nei’s genetic diversity matrix and nucleotide diversity, while Group I showed lower genetic diversity and heterozygosity (Table 2). The Nei’s. genetic diversity between the two groups was 0.0570, with group II showing higher diversity, likely due to the introduction of new genetic materials over the past two decades from different germplasm pools. This suggests that the introduction of novel germplasm, especially indeterminate cultivars after 2000, played an important role in increasing genetic diversity in group II.

Despite the higher Nei’s genetic diversity in group II compared to Group I, both Groups displayed lower genetic diversity compared to the 0.3863 reported by [10] for 77 Brazilian soybean genotypes using 35 SSR markers, and even lower compared to the Nei index of 0.6286 estimated in 100 Indian soybean varieties [37]. These differences may be attributed to the types of markers used and the number and origin of accessions evaluated in each study.

In similar way, the high LD decay values observed in this study also indicate genetic diversity loss during soybean improvement [43, 47]. This suggests that the Brazilian soybean has a high level of genomic homogeneity, resulting in increased LD and likely larger haplotype blocks. LD decay was higher in Group II compared to Group I, most likely, an increase in the size of the haplotype blocks. LD decay was higher in group II compared to group I, indicating greater homozygosity in group II and highlighting diversity as a key factor distinguishing Brazilian soybean cultivars. The LD decay distance observed in this study was higher than the results reported by [20], who studied 70% of the USDA Soybean Germplasm core collection and found that LD fell below 0.2 at approximately 250 kb. [45, 46] observed that LD reached r² = 0.1 after 574 kb among North American elite cultivars. As expected, [46] found that LD was more extensive in North American cultivars than in landraces and in landraces than in wild soybeans. The LD declined to half of its maximum value at 160 kb, 75 kb, and 20 kb for euchromatic regions, and 970 kb, 900 kb, and 350 kb for heterochromatic regions in North American landraces and wild cultivars, respectively. A recent study using 169 tropical soybean cultivars found LD declining below 0.2 at approximately 8.5 Mb [11], suggesting high genetic relatedness among tropical soybeans. Given the severe genetic bottleneck during soybean domestication, the high LD decay pattern observed in Brazilian cultivars was expected [9, 15, 46–49]. This loss in genetic diversity is common in crops that have been subjected to significant selection pressure during domestication and subsequent selective breeding [10, 47].

The number of linkage disequilibrium (LD) blocks observed is consistent with the high LD pattern and varies among different studies depending on the material set. For example, [20] found 5,607 haplotype blocks in a study of 18,489 *G*. *max* from the USDA Germplasm Collection with 42,509 SNPs, while [48] found 4,624, 5,226, and 3,093 haplotype blocks in 806 wild accessions, 5,396 landraces, and 562 North American cultivars, respectively. Consistent with our results, [11] found 941 linkage disequilibrium blocks in a study of 169 tropical soybean cultivars based on 3,780 SNPs from the BARCSoySNP6K.

### Sub-population structure in group I recovery the historical landmarkers of Brazilian breeding

We explored sub-structure variations within Group I, which contains 287 cultivars, to analyze the grouping patterns. We identified seven subpopulations; however, none had individuals exclusively from a single maturity group, stem termination type (STT), decade, or company. Nevertheless, we observed that cultivars with common ancestors or sharing similar traits, such as resistance genes for diseases prevalent in Brazil, were clustered together. This clustering reflects the increase in soybean production area and shifting cultivation from the South to the Central regions of Brazil [5–7].

In Subgroup 1, we found a cluster of some of the oldest Brazilian cultivars, such as IAC-2, Industrial (1967), IAC-4 (1975), Vicoja (1969), and Santa Rosa (1965), as well as the North American cultivar Pelicano (1951), introduced in the early 1950s. This subgroup also includes cultivars from the 1980s and 1990s, like BRS Seridó, BRS Sambaiba, and IAC-10 (1981), as well as some released after 2000.

Subgroup 2 is closely related to subpopulations 4 and 6, highlighting traditional sources of resistance, particularly to nematodes. Subgroup 2 includes cultivars such as BRSGO Chapadões, Ipameri, Raissa, BRSMT Pintado, BRSMG 250 [Nobreza], and BRS Jiripoca, released by Embrapa from the late 1990s and early 2000s, all resistant to soybean cyst nematode (SCN). This subgroup also includes six cultivars from Tropical Melhoramento Genético (TMG), released between 2006 and 2008, and four cultivars from Pioneer.

In Subgroup 4, we identified varieties resistant to root knot nematodes (RKN), such as Bragg (1966), BR 6-Nova Bragg, BRSGO-Luiziania (2000), BRS27 (Cariri), BRS 13 –Maravilha (1985), BRS Pioneira, BRS Valiosa, and BRS233, which are likely derived from the American cultivar Bragg introduced in the 1970s. Important RKN donors in this group include BRMG 46 Conquista (1995), BRSMG 68 Vencedora (1998), and BRSMG Garantia (2000).

Subgroup 5 contains a mix of Embrapa varieties and germplasm from other companies, including Monsanto, IAC, Coodetec, Pioneer, TMG, and FT Sementes, from the late 1990s and 2000s. Notably, the cultivar Paraná (1973) is closely related to the American cultivar Davis, a significant source of resistance to *Cercospora sojina*, which causes frogeye leaf spot. This fungus was first detected in Brazil during the 1970/1971 season and caused substantial yield losses throughout the 1970s and 1980s, particularly affecting cultivars like Bragg in Paraná and Doko in the Savanas region, which were not resistant at that time.

Subgroup 6 includes early cultivars with resistance to Southern stem canker, caused by *Diaporthe aspalati*, such as the American cultivar Tracy M, Embrapa-1 (1991), and IAS5 (1973). This disease was first detected in Brazil in the late 1980s, presenting significant challenges to soybean production. This group also includes the cultivar DOKO (1976), one of the first Brazilian cultivars adapted to low latitudes in central Brazil (Savanas) and showing a long juvenile period. Doko is close to Embrapa 20 and other Embrapa cultivars released in the 1980s, as well as varieties from Coodetec and Monsoy released in the 2000s.

In Subgroup 3, similar to Subgroup 4, a large number of cultivars from Embrapa are represented (approximately 75%), alongside three Monsanto (Bayer) cultivars released during the 1990s and 2000s, as well as some older cultivars like BR1, BR4, Ivaí, União, Ocepar 10, and FT Abyara from the 1970s and and 1980s.

The 7th cluster is the most dispersed across the tree, including 19 varieties from Nidera (Syngenta), Coodetec, Embrapa, Monsanto, TMG, FT Sementes, and also three Japanese varieties: Orba, Shiraniu, and Awashima Zairai. From the Nei’s Diversity and FST values matrix (Table 4), we observed that Subgroups 5 and 7 have the highest average Nei’s genetic distances to other subgroups, while Subgroup 4 has the lowest average Nei’s genetic distance. This suggests that Subgroups 5 and 7 are more genetically divergent from the other subgroups than Subgroup 4. The smallest Nei’s genetic distance is between Subgroups 5 and 6, indicating that these two subgroups are closely related genetically. The largest Nei’s genetic distance is between Subgroups 1 and 7, indicating the greatest genetic divergence among all Group I subgroups.

Regarding FST values, Subgroup 5 has the highest average FST value, while Subgroup 4 has the lowest. This suggests that there is greater population differentiation between Subgroup 5 and other subgroups compared to Subgroup 4. The smallest FST value is between Subgroups 6 and 7, indicating minimal population differentiation, while the largest FST value is between Subgroups 1 and 5, showing the greatest population differentiation among the seven subgroups. Although differences between subgroups exist, such as Subgroup 5 showing more diversity compared to Subgroup 4, the AMOVA analysis suggests that most of the genetic variation occurs within subgroups rather than between them.

### Regions under selection under 60 years of breeding in Brazilian germplasm

Domestication frequently causes a decrease in the genetic diversity, with genomic regions associated with agronomic traits and environmental adaptation undergoing greater selection pressure. Artificial selection during domestication and breeding can reduce genetic diversity and affect allele frequencies at various loci in the genome, potentially leading to fixation [16, 47]. These signatures of selection have been used many species, including soybean, to reveals breeding history and specific genomic regions responsible to traits of interest fixed among subgroups in the population [48]. In this study, we detect selective sweeps between two groups of Brazilian soybean cultivars that have somewhat related genetic background. Using BayeScan, we discovered 123 genomic regions spread across all 20 chromosomes that showed considerable signs of selection (S3 Table). These SNPs are not randomly distributed throughout the genome, but are located in the same LD blocks as 440 QTLs related to agronomic traits and adaptability to Brazilian conditions. We highlighted QTLs associated with resistance to important soybean diseases in Brazil, regional adaptation to short days and cycles, yield, and seed quality and composition. This provides strong evidence that the fixation of these loci was likely due to selection rather than random genetic drift.

Our study includes a large collection of Brazilian soybean varieties spanning six decades, allowing us to capture a substantial number of variations resulting from artificial selection during breeding, which are in LD with QTLs of agronomic traits. For the disease resistance category, we detected QTLs by selective sweeps in a region on chromosome 14, in LD block 1137, containing the resistance gene Rdm? for stem canker [49]. This block covers a region between positions 1,697,620 and 1,989,717 in the genome, where the resistance locus was previously mapped in a panel predominantly composed of Brazilian cultivars [49], confirming a fixed region in the Brazilian germplasm. The resistant allele is frequent in 69% of Group I and only 19% of Group II. Stem canker, caused by *Diaporthe aspalathi*, was first detected in Brazil in the late 1980s, causing significant constraints to soybean production [50]. Most cultivars released during 90s decade onward are reported to be resistant to stem canker. Recently, there is no report of significant occurrence of steam canker disease in Brazil, however, a lower frequency of the resistance locus in chromosome 14 was detected in Group II, which contains the majority of newer cultivars.

We also detected a significant number of QTLs related to resistance to soybean cyst nematode (SCN). We identified 13 QTLs previously described in the literature, distributed across 6 chromosomes, associated with SCN resistance, including chromosomes 18 and 8, where the main resistance genes *Rhg1* and *Rhg4* are located [51, 52]. Additionally, 5 QTLs were detected in chromosome 11, associated with the resistance to the races 2 and 5 derivate from PI90763, in a region where *Rgh*2 resistance gene was mapped [53]. In Brazil, SCN was found in soil samples from the States of Mato Grosso, Minas Gerais, and Mato Grosso do Sul collected after the 1991/92 growing season [54]). Since its identification, this disease has spread rapidly and now it is established in majority of soybean production regions of the country causing severe losses. A survey in 2006, identified at least 11 races of SCN with a higher concentration in the states of Mato Grosso, Goias, and Mato Grosso do Sul [55, 56], which are major soybean-producing areas in the country. The introduction of SCN resistance into Brazilian soybean germplasm began with the crossing of local germplasm with American varieties. Greater variability in resistance genes/QTLs could be expected in Brazilian varieties compared to North American ones. Unlike the USA, where PI88788 is the primary source of SCN resistance in commercial varieties, the main sources of SCN resistance in Brazil are Peking, PI88788, PI90763, Hartwig and PI437654.

In the category of Plant Development and growth, we highlighted QTLs related with yield, such as node number, pod number, plant height, interbranch length, leaflet area and chlorophyll content, seed yield and total seed number. These QTLs are distributed in 19 LD blocks in different chromosomes, including 11, 12 and 19, where important QTLs linked to yield were previously described [57]. In chromosome 11, we highlighted SNPs under selection in LD block 889, in an interval lower than 1 MB, where QTLs associated with maturity and yield traits such pod and node number, plant height and seed yield in early maturity soybean plant were detected [58]. A unique SNP under selection linked to this block is distributed high frequency (>90%) of the materials of Group II, and around of 70% of Group I. In chromosome 19, an interval of 2 Mb (43,386,606 to 45,510,542) contains at least five QTLs previously described as related with yield, in the LD blocks 1664, 1665 and 1667. In this region, main QTLs for pod number, leaflet width and area, plant height and Interbranch length are described [57, 58].

These QTLs may reflect years of artificial selection through yield increments. Since the beginning of the last decade, Brazil has been the 1st worldwide soybean producer, being responsible for around 35% of the global soybean production. Historical yield records suggest that soybean yields along the 1960–2021 period increased at a 22% higher rate in Brazil (39.4 kg ha^−1^ yr^−1^; 1.92% yr^−1^), as consequence of increase in area and yield [59]. The increase in yield has been reported in many studies. Todeschini et al. [60] showed an overall rate of genetic progress of 2.4% yr^−1^ from 1965 to 2011. Recently, Abdala et al [61] found seed yield genetic progress evident for soybean cultivars panel released after 2010 in different environments, in the high, medium, and low yielding environment, with relative rates of 0.9%, 1.0% and 0.5% yr^−1^, respectively. Moreover, genetic progress rates from 1966 to 2011 in Brazil were consistently different within regions, ranging from 0.33 to 0.42% yr^−1^ in the south and 0.47 to 0.77% yr^−1^ in the midwest [62].

Additionally, we also found 73 unique SNPs under selection linked to 48 QTLs distributed in 16 chromosomes related to the timing of flowering and maturity (first flower, reproductive period, and R8 full maturity). Reproductive phenology is one of the most important characters for maximizing soybean yields under various photoperiod and climate conditions. In addition to influencing time to flowering and maturation, most maturity genes and QTLs affect various agronomic traits that are dependent on reproductive development, such as grain yield and seed quality, as well as stress tolerance [62–65]. Prior to 1960, Brazilian soybean cultivars were mainly imported from the United States, and the cultivation areas were limited to south regions, at latitudes above 22°S [66]. This limitation was overcome by the introduction of the long juvenile (LJ) trait in the 1970s, allowing soybean production to expand to lower latitude (tropical) areas [66, 67]. The LJ trait features prolonged vegetative growth period under short day condition, allowing sufficient vegetative biomass to be produced to support a larger grain yield, which is an important adaptive trait at low latitudes [67].

At least 12 major genetic loci E1 to E11 and J, and several QTLs, such as Tof11/Gp11, Tof12/Gp1/qFT12‐1, and qDTFJ has been identified as involved in flowering and maturity in soybean [68]. Dominant alleles at E1, E2, E3, E4, E7, E8, and E10 confer late flowering, whereas dominant alleles at E6, E9, E11, and J confer early flowering, however they are strongly affected by daylengths. According to a previous study, cv. Williams 82 has the genotype “e1-as, E2, E3, E4, a northern US cultivar, more adapted to long days conditions.

We found SNPs under selection in the same LD block of the two major E genes, E1la (Glyma.04G156400) and E2 (Glyma.10G221500), and for the QTL tof12-1 (Glyma12G073900), which most differentiate Group I and II of Brazilian cultivars. The E1 (Glyma.06G207800) is the central hub of the flowering time in soybean. It encodes a transcription factor that inhibits flowering [69]. E1 has two homeologs in soybean, E1La and E1Lb [69, 70] with expression patterns similar to that of E1 under both LD (long day) and SD (short days). Both genes function as inhibitors of flowering, like E1 [70]. A recent study demonstrated that after J, E1 has the next most important effect on time to maturity in tropical environments [71, 72]. In our panel, the majority of the cultivars (309 out of 370), possess allele E1, diverging from Williams 82`s haplotype. The E1 allele has a high frequency in Group I (85%), and is present in almost all members from Group II (79 out of 83 accessions), being fixed on the Brazilian cultivars released after 2000`s years.

Similarly, E2 gene also promotes late flowering. It encodes a soybean ortholog of Arabidopsis GIGANTEA (GmGIa), which plays multiple roles in the circadian clock and flowering. The e2 allele and a loss‐of‐function mutant from the TILLING population encoding both a truncated protein shows early flowering phenotypes [73]. The SNP under selection S10_45670539, located in the LD block containing E2 gene, exhibits a dominant genotype compared with Williams 82 in 76 and 62% of accessions in Group I and II, respectively. Recently, Maldonato dos Santos et al. [74], comparing genetic bases between the Brazilian and US germplasm, also identified SNPs with high FST located on three known maturity loci: E1, E2 and FT2a, confirming the importance of E1 and E2 in adaptation to tropical conditions, as found in our analysis.

In contrast to the E1‐E4 loci, E6 and J are primarily involved in promoting flowering under SD. Recessive e6 or j alleles inhibit flowering and prolong both the vegetative and reproductive growth periods, contributing for LJ trait [75, 76]. Under SD, recessive j allele results in the upregulated expression of E1, contributing to late flowering [77]. We were unable to identify QTLs associated with J alleles distinguishing Groups I and II of Brazilian soybean. However, we found a QTL in the same LD block (947) of the Tof12 (Glyma.12G073900), which encodes the PSEUDO‐RESPONSE REGULATOR (PRR) 3, GmPRR3a and GmPRR3b [78–80]. GmPRR3a and GmPRR3b share 41% and 61% amino acid sequence identity with Arabidopsis PRR3, respectively. These genes are involved in circadian clock linked to flowering in soybean, and are described as exerting a positive effect on E1 expression, contributing to late flowering. Four SNPs under selection (ss715613160, ss715613154, ss715613148, ss715613171) were identified, with allele variants (A/G) present in 60% and 40% of accessions in Group I, respectively, while the A allele occurs in 84% of the accessions in Group II. These results reveal potential alleles in Brazilian germplasm contributing to adaptation to different regions. Lu et al, [81] also demonstrated that GmPRR3b is located in a selective sweep region showing evidence of selection during both soybean domestication and subsequent genetic improvement when they analyzed FST and the ratio of nucleotide diversity (qp) between three evolutionary populations: 58 wild accessions, 279 landraces; and 48 improved cultivars.

In this category, we also found SNPs under selection linked with SST. The SST was the most divergent trait between Groups I and II. We detected a QTL on chromosome 19 associated with stem shape, which is linked to eight SNPs under selection. Previous research has identified *Dt*1 gene as a causative gene for QTLs in this region [82]. The *Dt*1 gene was mapped to chromosome 19 [67, 68], within the haplotype block 1667 defined in our study. The region associated with *Dt*1 spans 218,465 bp. We found that 93% of genotypes in Group II carry the A allele, compared to only 7% in Group I. These findings support the information presented and highlight a significant characteristic that distinguishes between Groups I and II.

## Conclusions

The study aimed to investigate the genetic diversity and population structure of a historical collection of Brazilian soybean cultivars using high-throughput genotyping with SNP markers. The results showed a limited genetic background in the Brazilian germplasm due to multiple cycles of selection and recombination from a small number of USA cultivar ancestors. The population structure analysis detected two groups primarily divided by stem termination type and year of release, indicating the history of soybean expansion in Brazil.

LD analysis identified patterns that can affect molecular breeding strategies for selection of target loci for agronomic traits. The identification of 123 genomic regions under selection associated with 440 QTLs provides important information for future breeding programs to improve soybean productivity and adaptation to tropical conditions. Overall, the study provides valuable insights into the genetic diversity and population structure of Brazilian soybean cultivars and can serve as a basis for future breeding strategies.

## Supporting information

S1 Table Soybean accessions composing the panel. Maturity group (MG), stem termination type (SST) and year of release (YEAR) and assigned groups.
(XLSX)

S2 Table Analysis of molecular variance (AMOVA) evaluated with 19,576 SNPs. (XLSX)

S3 Table List of the SNPs under selection and associated to soybean QTLs. (XLSX)

S4 Table QTLs positions associated to the SNPs under selection. (XLSX)
